# New antifungal 4-chloro-3-nitrophenyldifluoroiodomethyl sulfone reduces the *Candida albicans* pathogenicity in the *Galleria mellonella* model organism

**DOI:** 10.1007/s42770-019-00140-z

**Published:** 2019-09-04

**Authors:** Monika Staniszewska, Małgorzata Gizińska, Michalina Kazek, Roberto de Jesús González-Hernández, Zbigniew Ochal, Héctor M. Mora-Montes

**Affiliations:** 1 Faculty of Chemistry, Warsaw University of Technology, Noakowskiego 3, 00-664 Warsaw, Poland; 2 KONDRAT&Partners, Al. Niepodległości 223/1, 02-087 Warsaw, Poland; 3 Laboratory of Physiology, The Witold Stefański Institute of Parasitology, Polish Academy of Science, Twarda 51/55, 00–818 Warsaw, Poland; 4 Departamento de Biologia, Division de Ciencias Naturales y Exactas, Campus Guanajuato, Universidad de Guanajuato, Noria Alta s/n, col. Noria Alta, C.P. 36050 Guanajuato, Gto. Mexico

**Keywords:** *C. albicans*, Virulence, Candidiasis, *G. mellonella*, Gene expression, Antifungal agent

## Abstract

*Candida albicans* represents an interesting microorganism to study complex host-pathogen interactions and for the development of effective antifungals. Our goal was to assess the efficacy of 4-chloro-3-nitrophenyldifluoroiodomethyl sulfone (named Sulfone) against the *C. albicans* infections in the *Galleria mellonella* host model. We assessed invasiveness of CAI4 parental strain and mutants: *kex2*Δ*/KEX2* and *kex2*Δ*/kex2*Δ in *G. mellonella* treated with Sulfone. We determined that *KEX2* expression was altered following Sulfone treatment in *G. mellonella-C. albicans* infection model. Infection with *kex2Δ/kex2Δ* induced decreased inflammation and minimal fault in fitness of larvae vs CAI4. Fifty percent of larvae died within 4–5 days (*P* value < 0.0001) when infected with CAI4 and *kex2Δ/KEX2* at 10^9^ CFU/mL; survival reached 100% in those injected with *kex2Δ/kex2Δ*. Larvae treated with Sulfone at 0.01 mg/kg 30 min before infection with all *C. albicans* tested survived infection at 90–100% vs *C. albicans* infected-PBS-treated larvae. Hypersensitive to Sulfone, *kex2Δ/kex2Δ* reduced virulence in survival. *KEX2* was down-regulated when larvae were treated with Sulfone: 30 min before and 2 h post-SC5314-wild-type infection respectively. *kex2Δ/kex2Δ* was able to infect larvae, but failed to kill host when treated with Sulfone. Sulfone can be used to prevent or treat candidiasis. *G. mellonella* facilitates studding of host-pathogen interactions, i.e., testing host vs panel of *C. albicans* mutants when antifungal is dosed.

## Introduction

Since incidences and frequency of the *Candida albicans* infections dominate over non-albicans species, there is still need to study the virulence of this pathogen, which represents an interesting window into the evolution of complex host-pathogen interactions and for the development of effective antifungal treatments [1–3]. The serine protease encoded by *KEX2* is among the *C. albicans*’ virulence factors that mediate its success as a pathogen. It is a representative of subtilisin family of proteins, processes enzymes, and its critical role in virulence was demonstrated previously [4–8]. As this approach still needs further analyses, here we continue studies assessing the *Galleria mellonella-C. albicans* infection model and the efficiency of novel antimicrobial agent. Recent studies [9–20] increased interest in the *G. mellonella* larvae as an alternative in vivo model due to the immunological and developmental similarities between insects and mammals. Therefore, the results obtained using these insects can serve as a starting point to study the *C. albicans* pathogenesis and generate hypotheses to be further tested in vertebrate models.

Since candidiasis is difficult to eradicate with the existing antimycotics [21], a new antifungal compound overcomes these deficiencies. We found [8] that the 4-chloro-3-nitrophenyldifluoroiodomethyl sulfone (named below Sulfone) treatment reduced the *C. albicans* pathogenicity in *G. mellonella*. It was shown [8] that the Sulfone displayed the minimal fungicidal concentration (MFC) against *C. albicans* at 0.25 μg/mL in in vitro studies and it was non-toxic against *G. mellonella* (lethal dose > 16 μg/mL). However, the impact of the Sulfone on metabolic pathways’ inhibition in *C. albicans* is not well understood yet. Our goals were to assess the efficacy of the Sulfone against the *C. albicans* infections in the *G. mellonella* host model and to compare the *KEX2* mutants’ susceptibility with the Sulfone in vivo. Additionally, we investigated whether the *KEX2* expression plays a role in the virulence of *C. albicans* in *G. mellonella*. The role of *KEX2* in virulence was tested by screening for attenuation in the *C. albicans* mutants. We determined in histopathological examinations whether the *KEX2* mutations affected the *C. albicans*’ tissue invasion capabilities. We examined if the *KEX2* expression is altered following the Sulfone treatment in the *G. mellonella-C. albicans* infection model.

## Material and methods

### 4-Chloro-3-nitrophenyldifluoroiodomethyl Sulfone’s synthesis

**Fig. 1:**
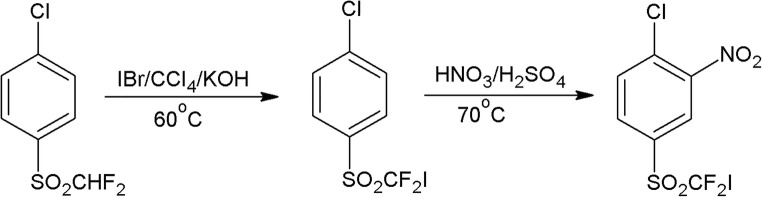
Synthesis of 4-chloro-3-nitrophenyldifluoroiodomethyl sulfone

4-Chloro-3-nitrophenyldifluoroiodomethyl sulfone was synthesized according to the scheme in Fig. 1, starting with the commercial 4-chlorophenyldifluoromethyl sulfone, which was iodinated through the reaction with iodine bromide, carried out in carbon tetrachloride with potassium hydroxide as a base [8]. The next step was nitration by fuming nitric acid and concentrated sulfuric acid.

### Strains culture, reagents, and growth conditions

*Candida albicans* strains used in the study are listed in Table 1. *C. albicans* cultures were grown in YEPD medium [24] at 30 °C overnight. Cultures were washed in sterile PBS and adjusted to the required cell density. YEPD medium supplemented with uridine at the final concentration of 50 μg/mL [25] was used when required. Transformants were selected on the MMD (0.7% wt/vol yeast nitrogen base without amino acids, 2% wt/vol glucose, 770 μg/mL Complete Supplement CSM-URA) liquid and agar (2%) media. The 5-FOA medium (4% wt/vol glucose, 0.2% wt/vol 5-fluoroorotic acid, 1.34% wt/vol yeast nitrogen base with amino acids, 4% wt/vol agar) was used when required.

**Table 1:** Strains used in the study

Species	Strain	Parental	Genotype	Reference
*C. albicans*	SC5314	None	Wild type	[22] (Gillum et al. 1984)
	CAI4	SC5314	*ura3Δ::imm434/ura3Δ::imm434*	[23] (Fonzi and Irwin 1993)
	*kex2Δ/KEX2*	CAI4	*ura3Δ::imm434/ura3Δ::imm434* *KEX2kex2*Δ*::*his*GURA3*his*G*	This work
	*kex2Δ/kex2Δ*	CAI4	*ura3Δ::imm434/ura3Δ::imm434* *kex2*Δ*::hisGkex2*Δ*::hisG*	This work

### Generation of the *C. albicans KEX2* mutant strains using the mini-ura-blaster technique [23]

The *C. albicans* strains attenuated in *KEX2* (Table 1) were constructed as follows: the two *KEX2* alleles in *C. albicans* CAI4 [23] were disrupted using the *URA3-dpl200* disruption cassette, that was PCR amplified from plasmid pDDB57 [25] using the specific primer pairs, containing complementary sequences to the 5′- and 3′-regions of the target ORF 19.4755 (Table 2). The transformants were generated in two rounds using the lithium acetate/single-stranded carrier DNA/PEG method as described in [25, 26] and the *URA3* marker was recycled by growing transformants on the 5-FOA medium. Briefly, in the first round of the transformation, we used primers (Table 2) amplifying plasmid DNA (*URA3-dp1200* disruption cassette) in PCR with 70 bp of *KEX2* flanking homology on either side and then transformed the PCR product into CAI4. The *URA*^+^ transformants grown on the MMD medium were screened with PCR that had undergone homologous integration at *KEX2*. Following this, the transformants were placed on 5-FOA to recycle the selection marker and use this strain in the second round of gene disruption. Due to high transformation frequencies (37 ± 5.7 CAI4 transformants/μg DNA [27]), the *Candida* integrity plasmid CIP10 was used as the positive control in the lithium acetate procedure. Consistent with this, CIP10 transformed *C. albicans* CAI4 [23] about 20 times more efficiently than the control plasmid (YPB–ADHpt).

**Table 2:** Oligonucleotides used in the study

Primers for *KEX2*	Sequence (5′ → 3′)
Forward primer for *KEX2* in first round	tttcaaatcactaatatattagattcttatctgtcatagaagatagaagttacaaccaacccacacatactgtggaattgtgagcggata
Reverse primer for *KEX2* in first round	aaacaatgcctttagggtatgtatcatttcttggtgtgtaggtctaataataataattattgtattgtatgttttcccagtcacgacgtt
Forward primer for *KEX2* in second round	ccaataaaattactaatatttatattgggatatttattatcaccaactttacaacaatatcaacaaattctgtggaattgtgagcggata
Reverse primer for *KEX2* in second round	aaactttcagcattaaaatcatcaaataatcgatctctagcttcatcttgttgtctatcgtattgtctttgttttcccagtcacgacgtt
Forward primer for *KEX2* screening	aagttacaaccaacccacacata
Reverse primer for *KEX2* screening	agggtatgtatcatttcttggtgt
Forward primer for *ACT1*	gacaatttctctttcagcactagtagtga
Reverse primer for *ACT1*	gctggtagagacttgaccaacca

### *Galleria mellonella* collection and treatment

The last instar larvae weighing between 180 and 250 mg were selected for the study. For killing assays, the *C. albicans* inoculum of 1 × 10^9^ cells per 10 μL of PBS was tested per larva. Larvae were incubated at 28 °C in 9-cm Petri dishes without food for up to 96 h post-infection and inspected every 24 h for survival. Since larvae deprived of nutrition demonstrated increased susceptibility to infection with the fungal pathogen *C. albicans* [28], we used the same conditions to show unequivocally the Sulfone activity/toxicity using the *C. albicans-G. mellonella* model. It is worthy to note that despite slight differences in the level of hemocytes and antimicrobial peptides between non- and starved larvae, their effectivity in killing *C. albicans* is not affected [28]. Since it was found that prophylaxis test is more likely to be successful because a compound is administered before infection, here we tested the Sulfone for either its prophylaxis or post-exposure activity [29]. Thus, we chose four time points to test the Sulfone dosing, e.g., 30 min and 1 h before infection (b. i.) with *C. albicans* as well as 30 min and 1 h post-infection (p. i.). In one experiment, 10 larvae per group were tested, where each one was injected with 10 μL of the Sulfone (at 0.01 mg/kg of larva) and with the *C. albicans* inoculum of 1 × 10^9^ cells per 10 μL of PBS (treated). The Sulfone and *C. albicans* inoculum were administered by injection into a different pro-leg using a Hamilton syringe. The Sulfone dose (0.01 mg/kg) was selected based on the previous studies [8] which proved that this dose is fungicidal against the *C. albicans* wild type and mutants (*KEX2/kex2∆* and *kex2∆/kex2∆*) in in vitro studies. Syringes were changed between treatments with different strains. Ten larvae without the *C. albicans* inoculum and 10 larvae inoculated with RPMI or PBS were included for control purposes. The larvae were incubated for 96 h in the above-described conditions. Host health index [30] of the treated larvae was assessed to rank the virulence of the *C. albicans* strains against *G. mellonella* [30]. Thus, larvae were monitored daily for the following attributes: activity and survival. Larvae were considered dead if they did not move after stimulation. Randomly selected live, non-melanized larvae, and treated larvae (each in triplicate) were suspended in 0.5 mL of PBS and mechanically disrupted (homogenized), then centrifuged at 100,000*g* for 1 h. Briefly, 100 μL of aliquots from the serial dilution (1/10,000,000) of supernatants were placed onto YEPD agar medium with penicillin (100 μg/mL) and incubated at 37 °C for 48 h. Thus, we confirmed the Sulfone’s fungicidal activity (MFC = 0.25 μg/mL) in vivo by comparison of the number of CFU of the *C. albicans* recovered from the larvae treated with the Sulfone and with the starting inoculum. The Sulfone’s antifungal activity was calculated using the formula: log reduction *R* = log CFU/mL control *Candida*, log CFU/mL *Candida* treated with the Sulfone, where *R* means the relative number of live fungal cells eliminated by the antifungal agent. To interpret the Sulfone activity in vivo, we adopted the criteria described by Majoros et al. [31], where fungicidal agent is that causing *a* ≥ 3 log reduction; i.e., after the treatment with the agent, the number of fungal cells is 1000 times smaller than the initial (control) number of fungal cells.

### Fungal cell staining with hematoxylin and eosin and periodic acid Schiff

Larvae were infected as described above. After 96 h of the maintenance of the larvae at 28 °C (see above), the surviving larvae from each group such as controls (PBS, RPMI, untreated) and those exposed to *Candida* and the Sulfone were anesthetized by chilling at − 20 °C for 18 h and then fixed, dehydrated, and stained, as described previously [8]. The stained tissues were observed using an Olympus FLUOROVIEW FV1000 confocal laser scanning microscope CLSM (Olympus, USA). Images were assembled using the Photoshop software (Adobe Photoshop CS3 Extended, France). Three larvae from each experimental group were used and the experiment was repeated on 3 independent occasions.

### RNA isolation and reverse transcription—quantitative polymerase chain reaction

Larvae were injected as described above. Three surviving larvae originated from each experimental group after 96 h, i.e., the larvae injected with *Candida* and Sulfone in time intervals as well as the untreated control group; they were frozen in liquid nitrogen and ground to powder with a mortar and pestle. The samples were homogenized and total RNA of *C. albicans* was isolated as described previously [32]. The total RNA from each sample was used in separate reverse transcription reactions with oligo dT. Total RNA from each sample (5 μL) was reverse transcribed with the Thermo Scientific RevertAid First Strand cDNA Synthesis Kit (K1691, Thermo Fisher Scientific, Waltham, MA USA). Each biological replicate was assayed for the targeted gene *KEX2* as well as the endogenous reference gene *ACT1* using the Thermo Scientific Maxima SYBER Green qPCR Master Mix (K0221, Thermo Fisher Scientific, Waltham, MA USA). The control groups were introduced to assess the influence of the RPMI Sulfone solvent and the lack of treatment on the *KEX2* expression. qPCR thermal cycling and fluorescent data acquisition were performed with a Light Cycler 96 Instrument (Roche Diagnostics GmbH, Germany/Roche Molecular Systems, Inc.) and Cq values were called using the Light Cycler 96 software. A 2^−*∆∆*Cq^ method [33] was then used to process these data to calculate the relative gene expression for the *KEX2* experiment. The experiment was performed in triplicate.

### Statistical analysis

Each experiment was performed at least in three replicates and the data were presented as mean values ± standard deviations (SD). Survival curves were plotted and differences in survival (logrank Mantel-Cox test and Gehan-Breslow-Wilcoxon test) were analyzed with the Kaplan-Meier Method using Graph Pad Prism 7 software (Inc.). *P* value < 0.05 was considered significant. Additionally, the variance ratio test (*F* test) was performed using MedCalc Statistical Software version 18.6 (MedCalc Software bvba, Ostend, Belgium; http://www.medcalc.org; 2018).

## Results

### Disruption of *KEX2* alleles reduces fungal injury during the host-fungus interaction

**Fig. 2:**
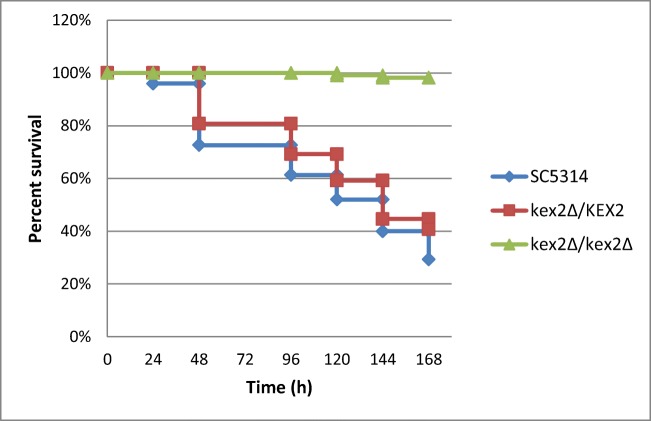
*G. mellonella* killing by different *C. albicans* strains depends on the *KEX2* gene disruption. The *kex2∆/KEX2* and *kex2∆/kex2∆* mutants along with the parental strain CAI4 were tested. PBS was used as a control. Groups of larvae from 110 to 150 per each strain were infected, as described in “Material and methods,” and survival was monitored every day. Survival experiment was performed at 28 °C. Larvae treated with PBS showed 100% survival in the tested period (data not presented). Statistics were calculated using Kaplan-Meier Graph Pad Prism 7 (logrank Mantel-Cox test, logrank test for trend, and Gehan-Breslow-Wilcoxon test), asterisks indicate significant differences, *P* value < 0.0001

**Fig. 3:**
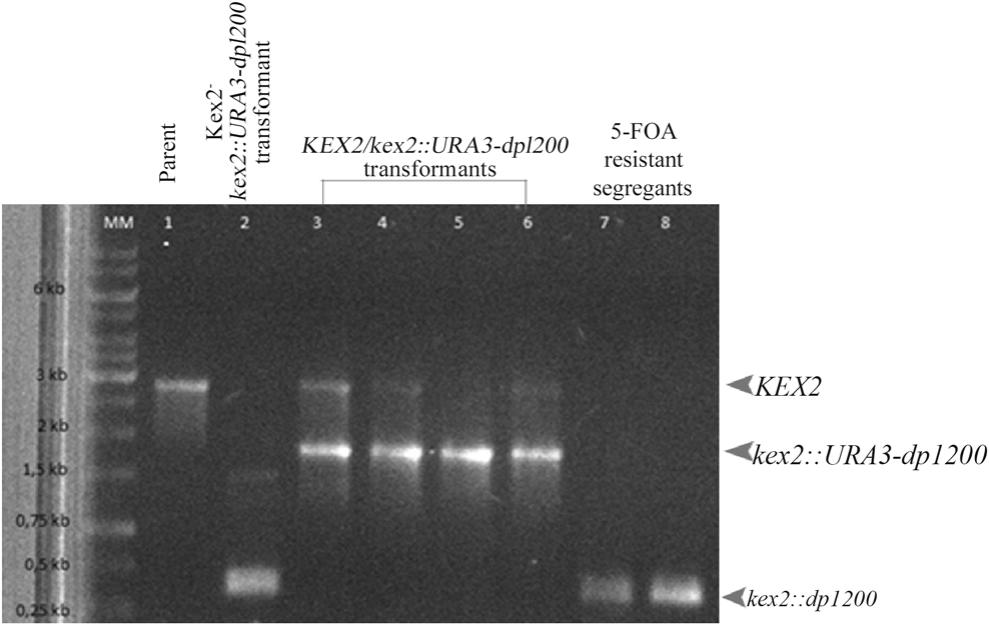
*C. albicans* transformants confirmed by PCR reaction using the screening primer pair (Table 2) amplifying the whole *KEX2* locus. Line: MM, marker; 1, CAI4 (*KEX2/KEX2*); 2, Kex2^−^*kex2::URA3dp1200* transformant; 3–6, KEX2/*kex2::URA3dp1200* transformants; 7–8, 5-FOA-resistant segregants

**Fig. 4:**
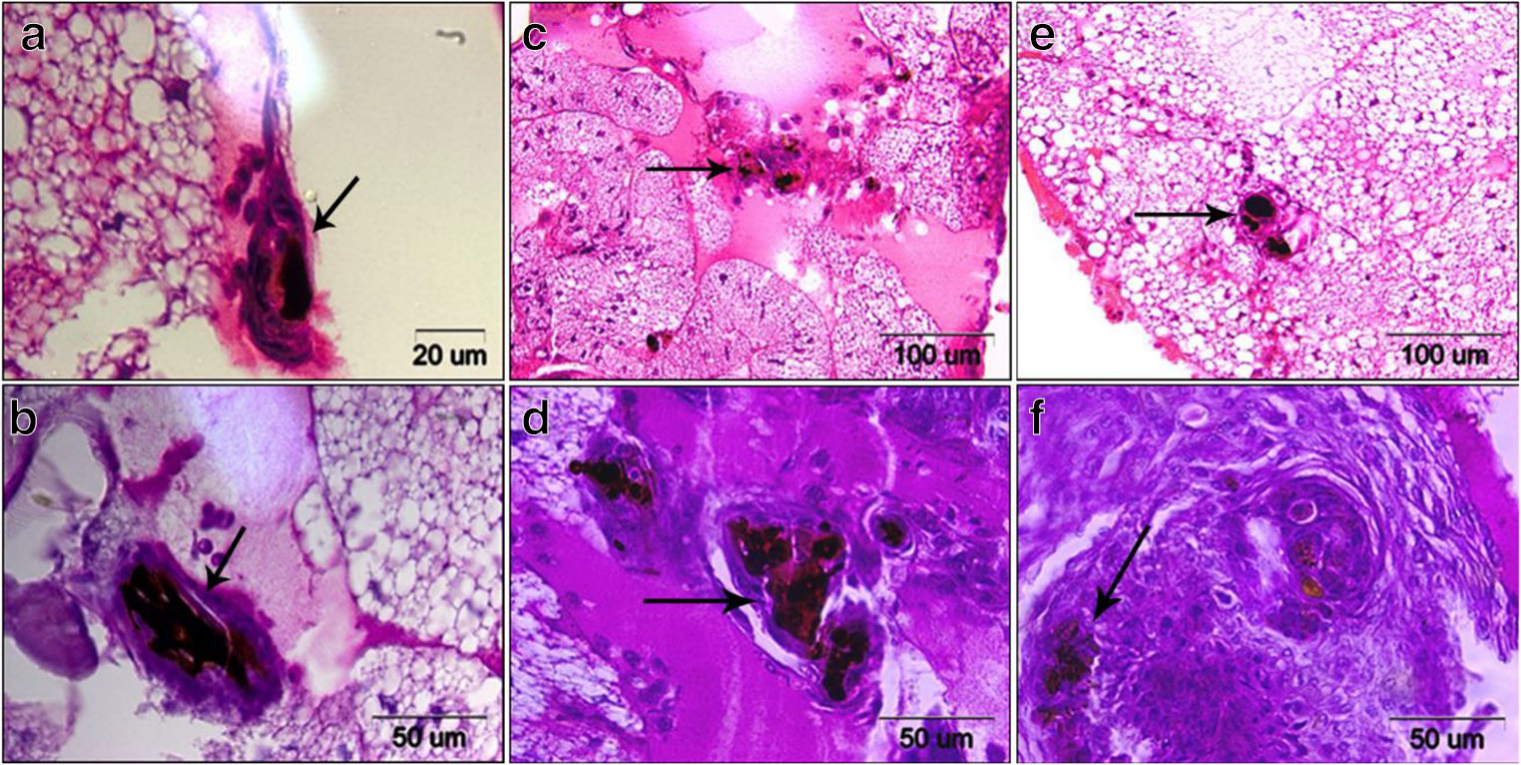
*Galleria mellonella* response to *C. albicans* infection. Histological analysis of larvae was performed using hematoxylin-eosin (HE) staining (**a**, **c**, **e**) and periodic acid Schiff (PAS) staining (**b**, **d**, **f**) at 6 days post-infection. **a**, **b** Larvae injected with CAI4. Fungal cells were isolated by hemocyte aggregation forming melanized nodules (arrows). **c**, **d***C. albicans kex2∆/KEX2* promotes hemocyte aggregation (arrows). **e**, **f** Small nodules (arrows) were formed in larvae infected with *kex2*Δ/k*ex2*Δ. Representative images are shown from histological analysis of 3 larvae per each strain per 3 independent experiments

Using the mini-ura-blaster technique, we disrupted *KEX2* to study its relevance in the *C. albicans* virulence in vivo. The electrophoresis results of the CAI4 parental strain and *KEX2* disruptants are presented in Fig. 2. The CAI4 strain (Ura^−^) with two deleted alleles of *URA3* was used as the parental strain (Fig. 2, line 1). The *KEX2* alleles were replaced by the *C. albicans URA3* gene flanked by direct repeats of the hisG sequence from *Salmonella typhimurium* (Fig. 2, lines 2–6). After CAI4 had been transformed with the URA blaster cassette, the Ura^+^ transformants were selected on uracil-deficient medium. The cells that had lost *URA3* by homologous recombination of the hisG sequences were selected on the medium containing 5-FOA because of its toxicity against the Ura^+^ cells (Fig. 2, lines 7–8). The larvae were infected with 1 × 10^9^ blastoconidia per larva of each strain: CAI4, *KEX2/kex2∆*, and *kex2∆/kex2∆*, and the survival was monitored (Fig. 3). Killing of the larvae depended on the strain injected. The larvae were killed significantly faster (*P* value< 0.0001) when infected with CAI4 and *kex2Δ/KEX2* at the inoculum density tested. Fifty percent of the larvae died within 4–5 days, while the survival still reached 100% in those injected with *kex2Δ/kex2Δ*, and after 6 days, all the latter larvae survived. The tissue sections were performed on the sixth day after the initiation of the fungal infection (Fig. 4). In the larvae treated with *kex2∆/kex2∆*, there were only very few infected areas compared with the larvae injected with CAI4 or *KEX2/kex2∆*. The larvae inoculated with *kex2∆/kex2∆* showed smaller nodules; these limited to the peripheral larval tissues. There was a relationship between the larvae death rate (Fig. 3) and progression to pathogenesis (mature nodules entrapping CAI4 or *KEX2/kex2∆* appeared, Fig. 3a–d). Therefore, we found evidence of the resistance of the larvae against the infection with *kex2∆/kex2∆* (Figs. 3 and 4e, f).

### The efficiency of the sulfone during the infection of larvae with *C. albicans*

**Fig. 5:**
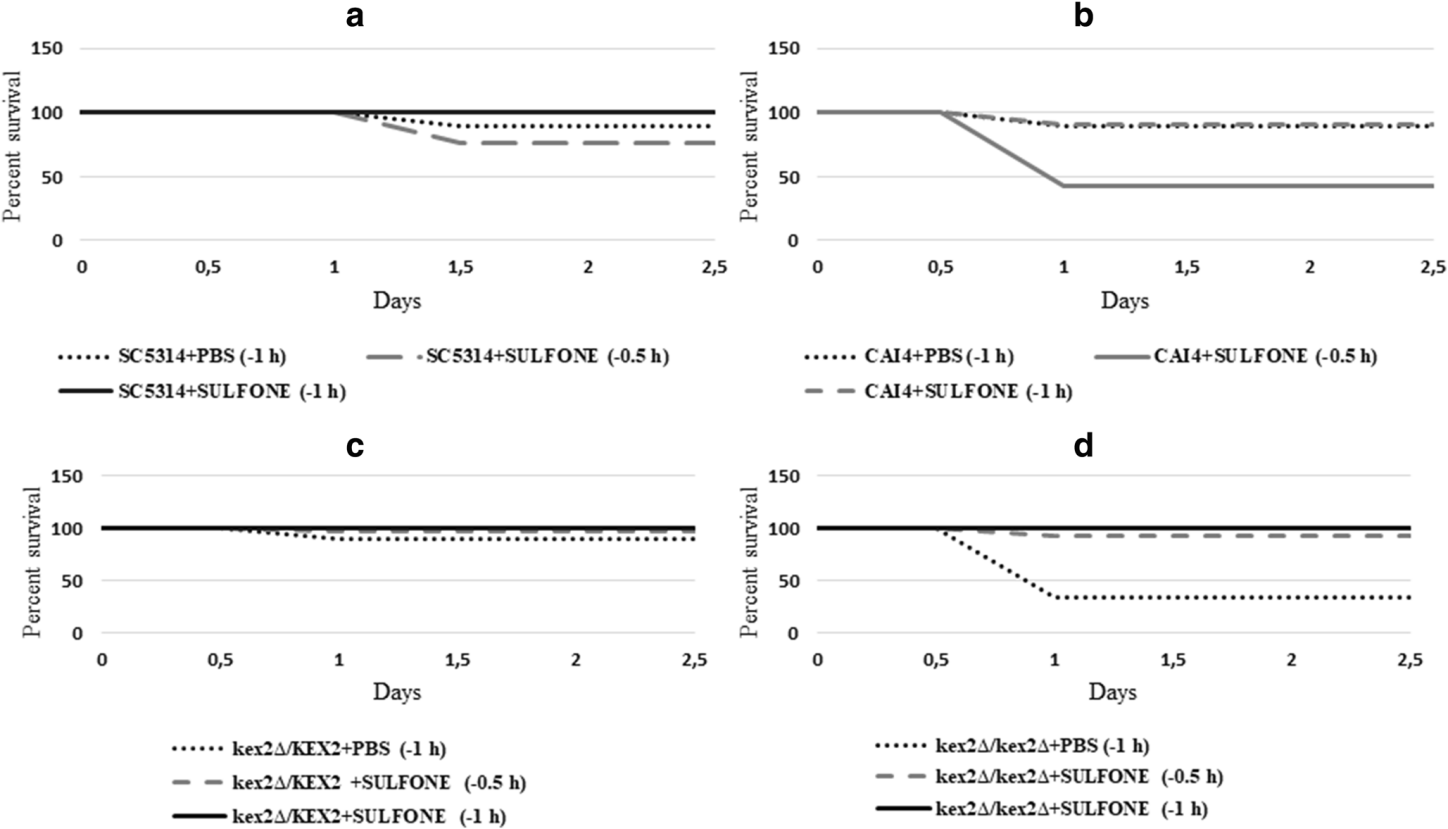
Effect of the Sulfone on *C. albicans* virulence in vivo Wax worms were injected with a dose of the Sulfone of 0.01 mg/kg in time intervals such as 1 h or 30 min before infection with *C. albicans* at 10^9^ CFU/mL. Kaplan-Meier survival curves of wax worms injected with **a** SC5314, **b** CAI4, **c***KEX2/kex2∆*, and **d***kex2∆/kex2∆*. Logrank (Mantel-Cox) test and Gehan-Breslow-Wilcoxon test: **a** survival proportion of SC5314 + PBS (− 1 h), SC5314 + SULFONE (− 30 min), and SC5314 + SULFONE (− 1 h) was 90, 75.862, and 100% respectively. Single asterisk indicates the survival curves sig different, *P* value = 0.0188. **b** Survival proportion of CAI4 + PBS (− 1 h), CAI4 + SULFONE (− 30 min), and CAI4 + SULFONE (− 1 h) was 90, 42.857, and 90% respectively. Double asterisks indicate the survival curves sig different, *P* value = 0.0002. **c** Survival proportion of *kex2∆/KEX2* + PBS (− 1 h), *kex2∆/KEX2* + SULFONE (− 30 min), and *kex2∆/KEX2* + SULFONE (− 1 h) was 90, 96.667, and 100% respectively. The survival curves are not sig different, *P* value = 0.2585. **d** Survival proportion of *kex2∆/kex2∆* + PBS (− 1 h), *kex2∆/kex2∆* + SULFONE (− 30 min), and *kex2∆/kex2∆* + SULFONE (− 1 h) was 33.333, 93.333, and 100% respectively. Triple asterisks indicate the survival curves sig different, *P* value < 0.0001

**Fig. 6:**
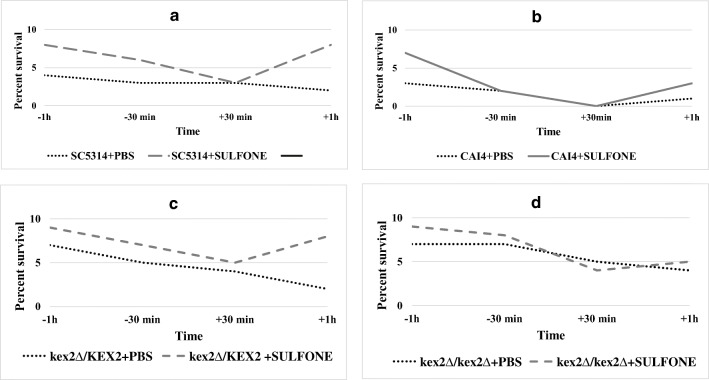
Effect of the Sulfone on larva health index. Treatment with the Sulfone at dose of 0.01 mg/kg per larva or PBS (control) was performed at time course such as 1 h or 30 min before infection with *C. albicans* (b. i.), 1 h or 30 min post-infection (p. i.) with *C. albicans*. **a**–**d** Variance ratio test (*F* test) of health index scores of wax worms was performed after 96-h incubation at 37 °C. In each graph, the differences between two curves (Sulfone vs PBS) were not significant (*P* value > 0.063)

We treated the larvae with the Sulfone at 0.01 mg/kg per larva separately in regiments as follows: 1 h or 30 min b. i. with *C. albicans*. The survival curves correlated with the strains and the Sulfone time course, with a higher death rate (57%) observed when the Sulfone was administered 30 min b. i. with CAI4 (Fig. 5b, *P* = 0.0002 vs PBS-treated group). The Sulfone-treated larvae 1 h b. i. with CAI4, *KEX2/kex2∆*, or *kex2∆/kex2∆* separately were protected against the development of candidiasis (Fig. 5b–d; *P* < 0.0001 vs PBS-treated group). Moreover, we evaluated the preventing effect of the Sulfone from the infection development when it was administered b. i. with *C. albicans*. Plating of the larval extracts provided evidence of the Sulfone’s antifungal activity in vivo (Table 3). We observed a reduction in the number of CFUs recovered from the Sulfone-treated larvae p. i. with *C. albicans* compared with the untreated larvae. In the Sulfone-treated larvae, the CFUs did not increase over the 6-day period as compared with the *C. albicans* control CFUs in the Sulfone-untreated larvae (Table 3). Collectively, the Sulfone increased the larval survival and caused 8.8-log reduction of CFUs when injected 1 h b. i. with CAI4 vs control. In contrast, the Sulfone inhibited the proliferation of *C. albicans kex2∆/KEX2* and *kex2∆/kex2∆* regardless of its administration (Table 3). Each infected larva was treated with the Sulfone at time course (b. i. and p. i.). The two major attributes such as activity and survival were assessed. The surviving larvae exhibited mobility after 96-h incubation. The wax worms injected with the Sulfone 1 h b. i. and p. i. with *C. albicans* showed a higher health index score (higher activity and survival) compared with those PBS-treated (Fig. 6, insignificant variation, *P* > 0.063).

**Table 3:** Fungicidal activity of the Sulfone (0.01 mg/mL) in the *G. mellonella* model in vivo at each time before infection (b. i)

Strains	Control *C. albicans* recovered from the untreated larvae CFU × 10^7^ ± SD	*C. albicans* recovered from the Sulfone-treated larvae CFU × 10^6^ ± SD		Logarithm reduction of *C. albicans* CFU recovered from the Sulfone-treated larvae log R*	
		30 min	1 h	30 min	1 h
CAI4	63 ± 2.5	3 ± 1.5	0 ± 0.0	2.3	8.8
*kex2∆/KEX2*	24 ± 4.5	0 ± 0.0	0 ± 0.0	8.4	8.4
*kex2∆/kex2∆*	13 ± 3.0	0 ± 0.0	0 ± 0.0	8.1	8.1

*Stands for decimal log reduction using the formula: log *R* = log CFU/mL control *Candida*, log CFU/mL *Candida* treated with the Sulfone

### The sulfone treatment modulates the *KEX2* expression in the *G. mellonella-C. albicans* infection model

*KEX2* was quantified as the relative gene expression data from each sample, and was normalized to the endogenous reference *ACT1* gene of *C. albicans* [32]. To evaluate the *KEX2* expression in the *C. albicans* treated with the antifungal Sulfone, we chose fungal inoculum at conc. of 10^9^ CFU/mL of PBS. Using the 2^−∆∆Cq^ method, it was shown that *KEX2* was slightly upregulated when the Sulfone at 0.01 mg/kg was dosed 30 min and 1 h p. i. with *C. albicans* SC5314 compared with the larvae injected only with the fungal inoculum of 10^9^ CFU/mL of PBS. During in vivo infection, *KEX2* was down-regulated when the larvae were treated with the Sulfone: 30 min b. i. and 2 h p. i. with SC5314 respectively (Table 4).

**Table 4:** Relative *KEX2* gene expression under the Sulfone in *G. mellonella* model

Treatment of *G. mellonella*	Sulfone (mg/kg)	Mean Cq *KEX2*		Mean Cq *ACT1*^REF^		∆∆Cq	2^−∆∆Cq^
		Average replicate	Std. dev.	Average replicate	Std. dev.	=(Cq_KEX2_-Cq_ACT1_)_treated_-(Cq_KEX2_-Cq_ACT1_)_control_	
Non-targeting control	Not applicable	35.24	0.37	27.72	1.21	–	1*
Injected with SC5314 at 10^9^ CFU/mL		34.66	0.51	28.44	1.12	− 1.3	0.70
Injected with Sulfone 30 min after post-infection (p. i.) with SC5314 (10^9^)	0.01	35.18	0.00	28.38	0.65	− 0.72	1.28
Injected with Sulfone 30 min before infection (b. i.) with SC5314 (10^9^)		35.18	0.00	29.02	0.00	− 1.3	0.64
Injected with Sulfone 2 h p. i. with SC5314 (10^9^)		32.81	0.00	27.20	1.24	− 1.91	0.09
Injected with Sulfone 1 h p. i. with SC5314 (10^9^)		35.83	2.40	27.52	1.64	0.79	1.73

All *G. mellonella* larvae were washed with 70% alcohol before examination; fungal RNA was isolated 6 days after treatment of larvae; 2^−∆∆Cq^, the data are presented as the fold change in the *KEX2* expression normalized to the reference *ACT1* and relative to the non-targeting control*

## Discussion

Besides *C. albicans*, kexin-like proteinases (Kex2) were identified in *Saccharomyces cerevisiae*, *Pichia pastoris*, and *C. glabrata* [34]. These Authors [34] showed that fungal Kex2 proteinases are similar in their substrate activities which have different functions according to the different biological backgrounds of the investigated fungi, including pathogenicity in humans. The *KEX2* gene from *C. glabrata* showed 51% and 62% identity and high structural similarities to its counterparts in *C. albicans* and *S. cerevisiae*. Bader et al. [35] revealed that Kex2 is involved in the processing of the proteins that are essential for cell surface integrity of *C. glabrata*. In *C. albicans*, Kex2 plays a role in the cell wall formation and interferes with aspartic proteases (Saps) in the cell wall’s remodeling mechanisms [4–7]. Moreover, the *C. albicans* cells attenuated in *KEX2* showed a defect in polarized growth and morphology [4–7]. Our in vivo results (lack of hyphae in histological analysis Fig. 4) are consistent with the in vitro findings of Newport and Agabian [5] that the double *KEX2* mutation affects morphogenesis. Furthermore, we are consistent with [5] showing that the *KEX2* disruption in *C. albicans* has a pleiotropic effect such as elevated sensitivity to the Sulfone. Our studies in *C. albicans* showed that the *KEX2* gene acts in its virulence process in vivo. It is a gene whose product processes enzymes which are critical for the virulence and ability of *C. albicans* to evade detection and destruction by the host’s immune system [4–7]. Bearing in mind that the understanding of the role of *KEX2* needs further investigation, we would like to emphasize below several observations that suggest its role during fungal challenge in the *G. mellonella* model. In contrast with the parental strain CAI4, among the larvae infected with the *kex2Δ/kex2Δ* mutant, 100% survived after day 5 (Fig. 3). Notably, the larvae infected with the relevant *kex2Δ/KEX2* mutant showed survival closer to the CAI4 parental strain (50% survived after day 4). This could be attributed to the absence of both of the *KEX2* alleles which are required for the attenuation the *C. albicans* virulence trait, while both alleles are not needed to maintain the *C. albicans*’ viability in vivo as shown here, and in vitro as previously reported [5]. Moreover, this phenomenon was described previously for other gene networks [36]. The larvae challenged with the *kex2Δ/kex2Δ* mutant displayed a decreased inflammatory response, as indicated by low nodule loads seen in the fat body (Fig. 4). Furthermore, this nodule-defective mutant displayed a minimal fault in fitness of the infected larvae. In contrast, the CAI4 parental cells exhibited abundant nodule formation comparable with *KEX2/kex2Δ* (Fig. 4). Interestingly, this consideration emphasizes further that the role of *KEX2* is more attributable to virulence in vivo. These data and the previous reports [18, 30] ruled out the ability of *C. albicans* to proliferate within hemocytes, leading to infection, and overwhelming the larval immune response.

On the basis of our previous study [8], we selected the Sulfone as the most promising lead candidate for further characterization in vivo. The findings presented in [8] and the current studies showed that administration of the Sulfone triggered immune response in larvae. We observed elevated hemocyte aggregation (Fig. 4) and fungicidal activity without the Sulfone’s negative influence on survival and pupation (Fig. 6). We included the Sulfone antifungal compound in our screen in order to identify whether it targets *KEX2*. The *kex2Δ/kex2Δ* mutant was hypersensitive to the Sulfone, and reduced virulence in the survival assay was noted (Fig. 5). As judged from our results and described for known antifungal drugs elsewhere [37, 38], complementation of other fungal genes can be successfully reduced by antifungal drugs when any one of them is deleted. In our study, this could be explained by the inability of other genes to substitute for the absence of *KEX2* in that strain when the Sulfone treatment is performed. Inhibition of other genes by the Sulfone is sufficient to prevent the development of infection.

Our study first involved identifying the expression of the responsive *KEX2* gene in vivo when treated with the Sulfone at 0.01 mg/kg per larva. The Sulfone affected the *C. albicans* cells’ proliferation in vivo in the time course tested, except for the Sulfone injected 30 min b. i. with SC5314 (Table 4). In this condition, the *KEX2* expression was almost unchanged, which indicates that the Sulfone both prevents the fungal cells’ proliferation and inhibits the virulence factor, thus it prevents the *KEX2* upregulation during infection. Targeting virulence is attractive in terms of antifungal drug development because it expands the extremely limited repertoire of targets in fungi [39]. Since the larvae injected with the CAI4 parental strain displayed lowered health index parameters compared with the relevant Sulfone-treated cells and mutants (Fig. 6), we hypothesized that the Sulfone targets *KEX2*. Due to the Sulfone’s action mode (inhibition of filamentation, adhesion, and biofilm formation, described in [8]), we decided to use a pretreatment modality to mimic its use in a prophylactic antifungal regimen starting 1 h and 30 min b. i. with *C. albicans*, thus maximizing the chances to detect any protective effect. The larvae which were administered the Sulfone 30 min b. i. with all the *C. albicans* tested survived the infection at 90–100%, this compared with the mortality observed in the infected and PBS-treated larvae (placebo control group in Fig. 6). We did not observe a perfect correlation between the two experiments, as measured in survival assays (Fig. 3 vs Fig. 6). For instance, the *kex2Δ/kex2Δ* mutant was less virulent in the untreated larvae (either with the Sulfone or PBS) than in the PBS-treated ones 1 h b. i. with this mutant, yet the larvae displayed a similar fungal load (Fig. 6). One possible explanation is that for the infected larvae, additional stress related to the injection with PBS or the cuticle piercing can be deadly; the larvae started dying in large proportions already on day 1. Thus, the death rate may be misleading, with an error introduced by the PBS injection: the infected and PBS-treated larvae yielded higher mortality, whereas the infected but PBS-untreated ones survived. Thus, a definitive conclusion will be revealed when this experiment has been repeated much more extensively than in the preliminary studies reported here. As shown by Ignasiak and Maxwell [40], sodium chloride is not toxic against *G. mellonella*, but the cuticle piercing with a needle as such can be traumatic for the larvae. Thus, in each step in the study, three following control groups: untreated, traumatized, and buffer-injected control ought to be included [40]. These Authors [40] found that although the *G. mellonella* larvae cannot fully replace the mammalian models, they provide the statistical robustness which animal models lack. Moreover, the antibiotic doses recommended for use in humans can be effective in systemic infections in the larvae, and the acute toxicity of compounds in wax moth larvae correlate to the toxicity in mice and rats.

Collectively, since the *C. albicans* virulence involves a complex regulatory network of genes, the presence of overlapping genes could mask any detectable phenotype due to an altered expression of other genes in the *kex2Δ/kex2Δ* cells. However, our results supported the well-established Sulfone’s anti-virulence activity [8].

Our data of the Sulfone revealed striking observations: *kex2Δ/KEX2* and *kex2Δ/kex2Δ* were less virulent when the Sulfone was administered 1 h or 30 min b. i. of the larvae. The *C. albicans* parental strain’s inoculum of 10^9^ CFU/mL of PBS induced the larval mortality at 50% compared with that those infected with the *C. albicans kex2Δ/kex2Δ* mutant. This mutant was able to infect the larvae but failed to kill the host cells when treated with the Sulfone. Moreover, treating the larvae with the Sulfone 30 min or 1 h b. i. with SC5314 prevented further *C. albicans*’ growth and effectively prevented the larvae from death. In conclusion, our novel findings described in this article suggest that the Sulfone can be used in direct therapy to prevent or treat potentially fatal fungal infections. Since no animal or animal-derived model of infection completely replicates human diseases [37], the *G. mellonella* systemic candidiasis model that we used here facilitates the host-pathogen interactions, i.e., testing a host vs a panel of *C. albicans* mutants when a new antifungal agent is dosed.

## References

[1] Kragelund C (2017). Exploiting new knowledge of Candidal infection for future antifungal combat. Oral Dis.

[2] Böhm L, Torsin S, Tint SH, Eckstein MT, Ludwig T, Pérez JC (2017). The yeast form of the fungus *Candida albicans* promotes persistance in the gut of gnobiotic mice. PLoS Pathog.

[3] Romo JA, Pierce CG, Chaturvedi AK, Lazzell AL, McHardy SF, Saville SP, Lopez-Ribot JL (2017). Development of anti-virulence approaches for candidiasis *via* a novel series of small-molecule inhibitors of *Candida albicans* filamentation. mBio.

[4] Bresnahan PA, Leduc R, Thomas L, Thorner J, Gibson HL, Brake AJ, Barr PJ, Thomas G (1990). Human fur gene encodes a yeast *KEX2*-like endoprotease that cleaves pro-beta NGF *in vivo*. J Cell Biol.

[5] Newport G, Agabian N (1997). *KEX2* influences *Candida albicans* proteinase secretion and hyphal formation. J Biol Chem.

[6] Newport G, Kuo A, Flattery A, Gill C, Blake JJ, Kurtz MB, Abruzzo GK, Agabian N (2003). Inactivation of Kex2p diminishes the virulence of *Candida albicans*. J Biol Chem.

[7] Holyoak T, Kettner CA, Petsko GA, Fuller RS, Ringe D (2004). Structrural basis for differences in substrate selectivity in Kex2 and furin protein convertases. Biochemistry.

[8] Staniszewska M, Bondaryk M, Kazek M, Gliniewicz A, Braunsdorf C, Schaller M, Mora-Montes HM, Ochal Z (2017). Effect of serine protease *KEX2* on *Candida albicans* virulence under halogenated methyl sulfones. Future Microbiol.

[9] Cotter G, Doyle S, Kavanagh K (2000). Development of an insect model for the in vitro pathogenicity testing of yeasts. FEMS Immunol Med Microbiol.

[10] Mowlds P, Kavanagh K (2008). Effect of pre-incubation temperature on susceptibility of *Galleria mellonella* larvae to infection by *Candida albicans*. Mycopathologia.

[11] Fuchs BB, O’Brien E, Khoury JB, Mylonakis E (2010). Methods for using *Galleria mellonella* as a model host to study fungal pathogenesis. Virulence.

[12] Kelly J, Kavanagh K (2010). Proteomic analysis of proteins released from growth-arrested *Candida albicans* following exposure to caspofungin. Med Mycol.

[13] Kelly J, Kavanagh K (2011). Caspofungin primes the immune response of the larvae of *Galleria mellonella* and induces a non-specific antimicrobial response. J Med Microbiol.

[14] Brown N, Heelan M, Kavanagh K (2013). An analysis of the structural and functional similarities of insect hemocytes and mammalian phagocytes. Virulence.

[15] Li DD, Hu GH, Zhao LX, Hu DD, Jing YY, Wang Y (2013). Using *Galleria mellonella*-*Candida albicans* infection model to evaluate antifungal agents. Biol Pharm Bull.

[16] Ullah I, Khan AL, Ali L, Khan AR, Wagas M, Lee IJ, Shin JH (2014). An insecticidal compound produced by an insect-pathogenic bacterium suppresses host defenses through phenoloxidase inhibition. Molecules.

[17] Champion OL, Wagley S, Titball RW (2016). *Galleria mellonella* as a model host for microbiological and toxin research. Virulence.

[18] Tsai CJY, Loh JMS, Proft T (2014). *Galleria mellonella* infection models for the study of bacterial diseases and for antimicrobial drug testing. Virulence.

[19] Rossi SA, Trevijano-Contador N, Scorzoni L, Mesa-Arango AC, de Oliveira HC, Werther K, de Freitas RT, Mendes-Giannini MJ, Zaragoza O, Fusco-Almeida AM (2016). Impact of resistance to fluconazole on virulence and morphological aspects of *Cryptococcus neoformans* and *Cryptococcus gattii* isolates. Front Microbiol.

[20] Sowa-Jasiłek A, Zdybicka-Barabas A, Staczek S, Wydrych J, Skrzypiec K, Mak P, Deryło K, Tchórzewski M, Cytryńska M (2016). *Galleria mellonella* lysozyme induces apoptotic changes in *Candida albicans* cells. Microbiol Res.

[21] Gizińska M, Staniszewska M, Ochal Z (2019). Novel sulfones with antifungal properties: antifungal activities and interactions with *Candida* spp. virulence factors. Mini-Rev Med Chem.

[22] Gillum AM, Tsay EYH, Kirsch DR (1984). Isolation of the *Candida albicans* gene for orotidine-5′-phosphate decarboxylase by complementation of *S. cerevisiae* ura3 and *E. coli* pyrF mutations. Mol Gen Genet.

[23] Fonzi WA, Irwin MY (1993). Isogenic strain construction and gene mapping in *Candida albicans*. Genetics.

[24] Ness F, Prouzet-Mauleon V, Vieillemard A, Lefebvre F, Noël T, Crouzet M, Doignon F, Thoraval D (2010). The *Candida albicans* Rgd1 is a RhoGAP protein involved in the control of filamentous growth. Fungal Genet Biol.

[25] Wilson RB, Davis D, Enloe BM, Mitchell AP (2000). A recyclable *Candida albicans URA3* cassette for PCR product-directed gene disruptions. Yeast.

[26] Wilson RB, Davis D, Mitchell AP (1999). Rapid hypothesis testing with *Candida albicans* through gene disruption with short homology regions. J Bacteriol.

[27] Murad AM, Lee PR, Broadbent ID, Barelle CJ, Brown AJ (2000). Cip10, an efficient and convenient integrating vector for *Candida albicans*. Yeast.

[28] Banville N, Browne N, Kavanagh K (2012). Effect of nutrient deprivation on the susceptibility of *Galleria mellonella* larvae to infection. Virulence.

[29] Kocisko DA, Caughey B, Kheterpal J, Wetzel R (2006). Cell-based PrP-res assay and in vivo scrapie testing. Methods in enzymology.

[30] Loh JM, Adenwalla N, Wiles S, Proft T (2013). *Galleria mellonella* larvae as an infection model for group A *Streptococcus*. Virulence.

[31] Majoros L, Kardos G, Szabó B, Sipiczki M (2005). Caspofungin susceptibility testing of *Candida inconspicua*: correlation of different methods with the minima fungicidal concentration. Antimicrob Agents Chemother.

[32] Staniszewska M, Bondaryk M, Malewski T, Schaller M (2014). The expression of the *Candida albicans* gene *SAP4* during hyphal formation in human serum and in adhesion to monolayer cell culture of colorectal carcinoma Caco-2 (ATCC). Cent Eur J Biol.

[33] Livak KJ, Schmittgen TD (2001). Analysis of relative gene expression data using real-time quantitative PCR and the 2(-Delta Delta C(T)) method. Methods.

[34] Bader O, Krauke Y, Hube B (2008). Processing of predicted substrates of fungal Kex2 proteinases from *Candida albicans*, *C. glabrata*, *Saccharomyces cerevisiae* and *Pichia pastoris*. BMC Microbiol.

[35] Bader O, Schaller M, Klein S, Kukula J, Haack K, Muhlschlegel F, Korting HC, Schafer W, Hube B (2001). The *KEX2* gene of *Candida glabrata* is required for cell surface integrity. Mol Microbiol.

[36] Khandelwal NK, Kaemmer P, Förster TM, Singh A, Coste AT, Andes DR, Hube B, Sanglard D, Chauhan N, Kaur R, d’Enfert C, Mondal AK, Prasad R (2016). Pleiotropic effects of the vacuolar ABC transporter MLT1 of *Candida albicans* on cell function and virulence. Biochem J.

[37] Talibi D, Raymond M (1999). Isolation of a putative *Candida albicans* transcriptional regulator involved in pleiotropic drug resistance by functional complementation of a pdr1 pdr3 mutation in *Saccharomyces cerevisiae*. J Bacteriol.

[38] Pais P, Costa C, Cavalheiro M, Romão D, Teixeira MC (2016). Transcriptional control of drug resistance, virulence and immune system evasion in pathogenic fungi: a cross-species comparison. Front Cell Infect Microbiol.

[39] Pierce CG, Uppuluri P, Tummala S, Lopez-Ribot JL (2010). A 96 well microtiter plate-based method for monitoring formation and antifungal susceptibility testing of *Candida albicans* biofilms. J Vis Exp.

[40] Ignasiak K, Maxwell A (2017). *Galleria mellonella* (greater wax moth) larvae as a model for antibiotic susceptibility testing and acute toxicity trials. BMC Res Notes.

